# Crystal structure of (*E*)*-N*’-[1-(4-amino­phen­yl)ethyl­idene]-2-hy­droxy-5-iodo­benzohydrazide methanol monosolvate

**DOI:** 10.1107/S2056989018008204

**Published:** 2018-06-08

**Authors:** Cong Nguyen Tien, Huong Le Thi Thu, Thin Nguyen Van, Trung Vu Quoc, Manh Vu Quoc, Thang Pham Chien, Luc Van Meervelt

**Affiliations:** a Faculty of Chemistry, Ho Chi Minh City University of Education, 280 An Duong Vuong Street, District No. 5, Ho Chi Minh City, Vietnam; b School of Natural Sciences Education, Vinh University, 182 Le Duan St., Vinh City, Vietnam; c Faculty of Chemistry, Hanoi National University of Education, 136 Xuan Thuy, Cau Giay, Hanoi, Vietnam; d Faculty of Foundation Science, College of Printing Industry, Phuc Dien, Bac Tu Liem, Hanoi, Vietnam; e Department of Chemistry, Hanoi University of Science, 19 Le Thanh Tong Street, Hai Ba Trung District, Hanoi, Vietnam; f Department of Chemistry, KU Leuven, Biomolecular Architecture, Celestijnenlaan 200F, Leuven (Heverlee), B-3001, Belgium

**Keywords:** crystal structure, *N*-substituted hydrazide, salicylic acid, Hirshfeld surface

## Abstract

In the title compound, C_15_H_14_IN_3_O_2_·CH_3_OH, two aromatic rings are linked by an *N*-substituted hydrazide function. The dihedral angle between the aromatic rings is 10.53 (8)°. The stereochemistry about the imine function is *E*. The methanol mol­ecule forms an O—H⋯O hydrogen bond to the hydrazide O atom. In the crystal, chains of mol­ecules running along the *c*-axis direction are formed by O—H⋯O hydrogen bonds. Adjacent chains are linked through N—H⋯O hydrogen bonds and π–π stacking inter­actions. The inter­molecular inter­actions in the crystal packing were investigated using Hirshfeld surface analysis, which indicated that the most significant contacts are H⋯H (38.2%), followed by C⋯H/H⋯C (20.6%), O⋯H/H⋯O (11.1%) and I⋯H/H⋯I (9.7%).

## Abstract 2

The synthesis and crystal structure of a new *N*-substituted hydrazide are reported. In the crystal packing, O—H⋯O and N—H⋯O hydrogen bonds predominate together with π–π stacking inter­actions.

## Chemical context   

*N*-substituted hydrazides have been attracted much attention for their structures, coordination ability, biological activities and transformations to heterocyclic compounds (Majumdar *et al.*, 2014 ▸; Asif & Husain, 2013 ▸; Khan *et al.*, 2017 ▸). Derivatives of salicylic acid act as anti­bacterial (Kumar *et al.*, 2012 ▸; Cui *et al.*, 2014 ▸; Sarshira *et al.*, 2016 ▸), anti­fungal (Wodnicka *et al.*, 2017 ▸; Abbas *et al.*, 2017 ▸) and anti­tumor (Murty *et al.*, 2014 ▸) agents. In addition, some salicylhydrazones exhibit significant anti­trypanosomal activity with IC_50_ ranging from 1 to 34 µM. *N*-substituted hydrazides containing the typical –C(O)—NH—N=C< functional group can be prepared by a condensation reaction between a hydrazide and a carbonyl compound (an aldehyde or a ketone).
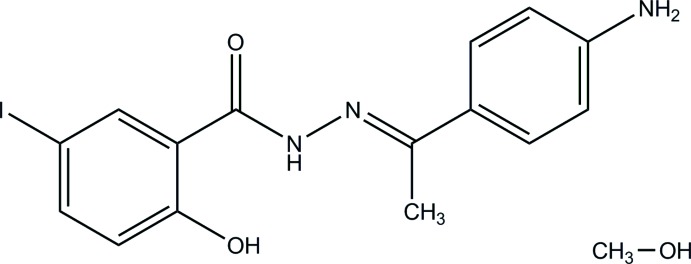


As a continuation of our research work to synthesize derivatives of 5-iodo­salicylohydrazide (Nguyen *et al.*, 2012 ▸), the new compound (*E*)*-N*’-[1-(4-amino­phen­yl)ethyl­idene]-2-hy­droxy-5-iodo­benzohydrazide methanol monosolvate was synthesized. The structure of the compound was determined by IR, ^1^H NMR, ^13^C NMR and HR–MS spectroscopy as well as X-ray diffraction and the crystal structure is reported herein.

## Structural commentary   

**Figure 1:**
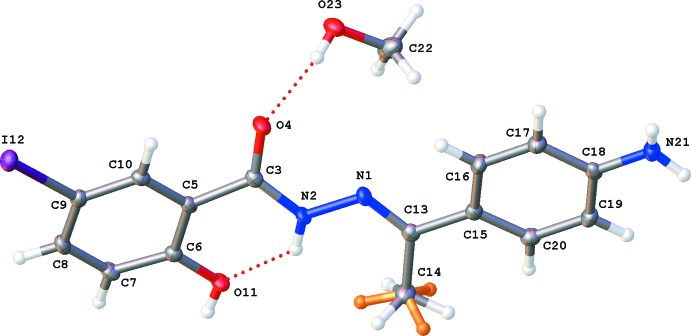
View of the asymmetric unit of the title compound, showing the atom-labelling scheme. Displacement ellipsoids are drawn at the 50% probability level. H atoms are shown as small circles of arbitrary radii. Intra- and inter­molecular hydrogen bonds are shown as dashed lines.

The title compound (Fig. 1 ▸) crystallizes as a methanol monosolvate in the monoclinic space group *P*2_1_/*c* with one hydrazide mol­ecule and a methanol solvate mol­ecule in the asymmetric unit. The OH group of methanol is hydrogen bonded to the hydrazide oxygen atom O4 (Fig. 1 ▸, Table 1 ▸). The dihedral angle between the aromatic rings is 10.53 (8)°. This relatively planar character of the mol­ecule is caused by an intra­molecular hydrogen bond, N2—H2⋯O11 (Table 1 ▸), and the presence of the hydrazide functional group and the C13=N1 double bond. The r.m.s. deviation from a plane through all 21 non-Hatoms is 0.291 Å [with a maximum deviation of 0.838 (1) Å observed for atom O4]. The torsion angles about the bonds of the hydrazide link between the two aromatic rings are: C15—C13=N1—N2 = −175.48 (15)°, C13=N1—N2—C3 = 178.71 (16)° and N1—N2—C3—C5 = −172.18 (15)°. The stereochemistry about the imine function C13=N1 is *E*. The planar character causes short contacts for the H atoms of methyl group C14 with the H atoms on atoms N2 and C20. As a consequence, this methyl group displays rotational disorder with occupancies of 0.66 (2) and 0.34 (2).

## Supra­molecular features   

**Figure 2:**
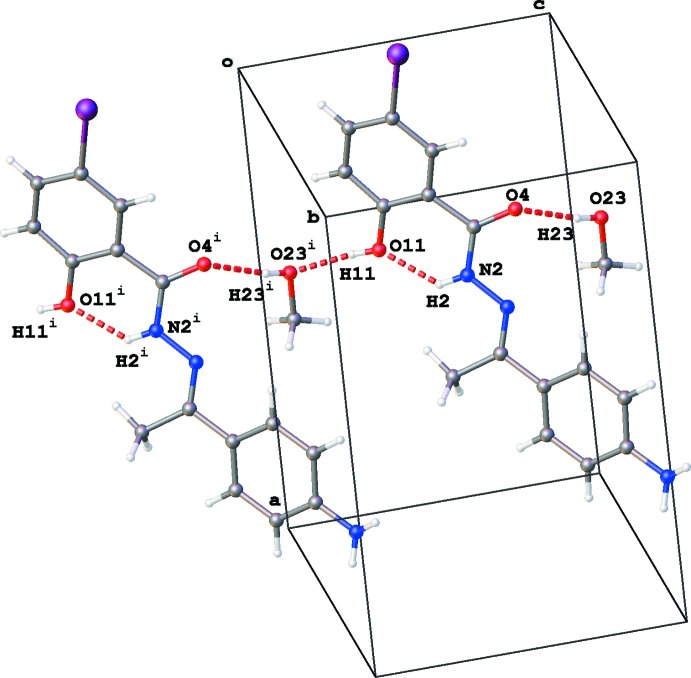
Part of the crystal packing of the title compound, showing the chain along the *c*-axis direction formed by O—H⋯O hydrogen-bonding inter­actions [see Table 1 ▸; symmetry code: (i) *x*, *y*, *z* − 1]. Only the major component of the disordered methyl group C14 is shown.

**Figure 3:**
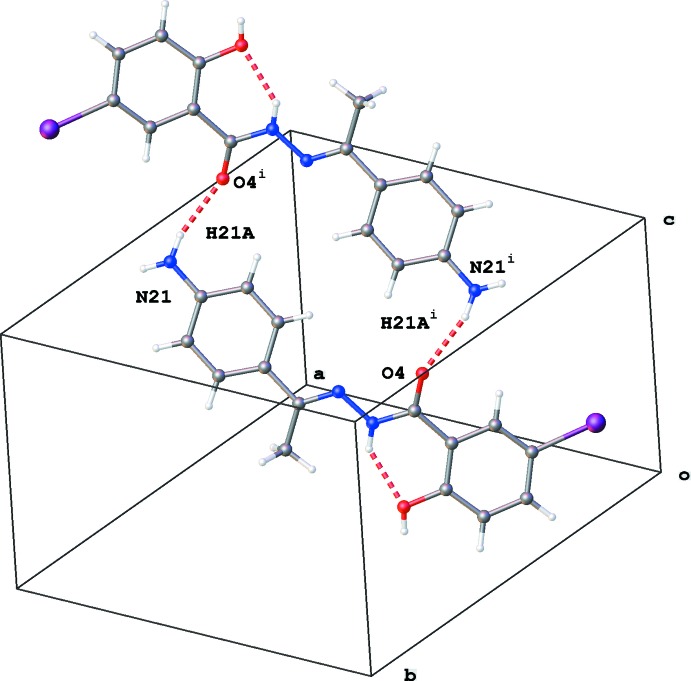
Ring of graph-set motif 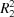
(22) formed by N—H⋯O hydrogen-bonding inter­actions [see Table 1 ▸; symmetry code: (i) *x* − 1, *y* − 1, *z* − 2].

**Figure 4:**
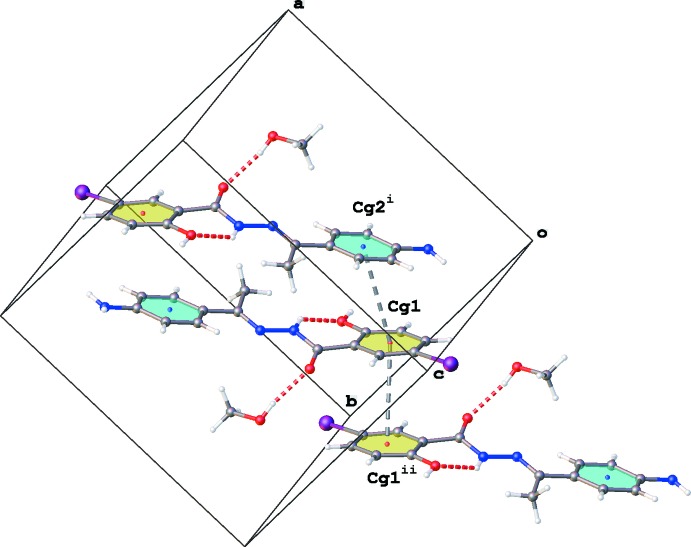
Part of the crystal packing of the title compound, showing the π–π stacking inter­actions between the amino­phenyl (blue) and iodo­phenyl (yellow) rings [symmetry codes: (i) −*x* + 1, −*y* + 1, −*z* + 1; (ii) −*x*, −*y* + 1, −*z* + 1].

In the crystal, chains of mol­ecules are formed along the *c*-axis direction by alternating O11—H11⋯O23^i^ and O23—H23⋯O4 hydrogen bonds (Table 1 ▸ and Fig. 2 ▸). The inter­action of adjacent chains through N21—H21*A*⋯O4^ii^ hydrogen bonds results in the formation of dimers with graph set 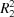
(22) (Table 1 ▸ and Fig. 3 ▸). Both aromatic rings are involved in π–π stacking inter­actions [*Cg*1⋯*Cg*1^i^ = 3.9769 (10) Å, slippage 2.042 Å and *Cg*1⋯*Cg*2^ii^ = 3.8635 (11) Å, slippage 1.596 Å; *Cg*1 and *Cg*2 are the centroids of rings C5–C10 and C15–C20, respectively; Fig. 4 ▸]. The crystal packing contains no voids.

**Figure 5:**
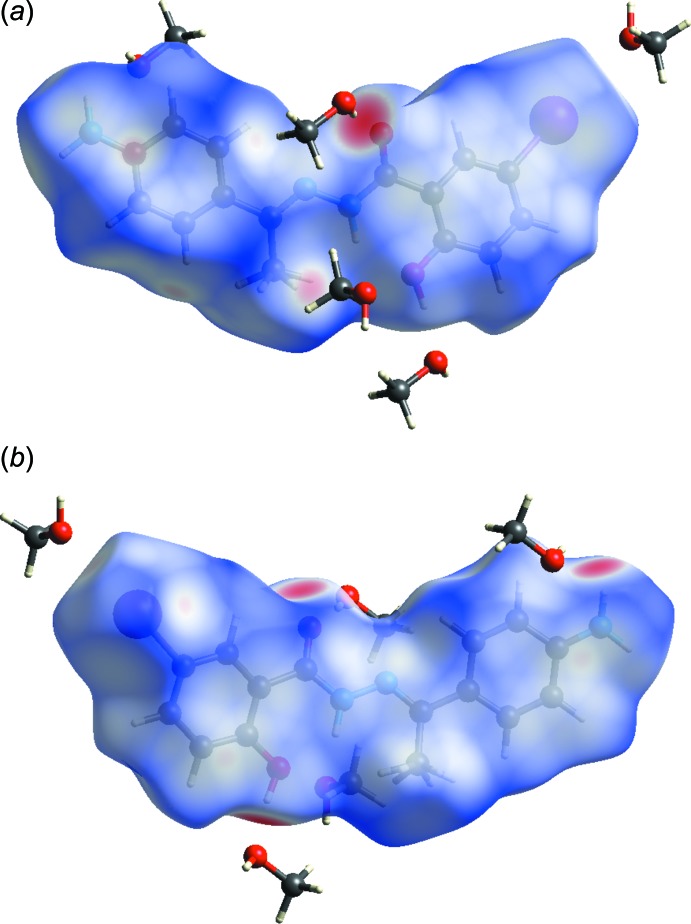
Views of the Hirshfeld surface for the title compound mapped over *d*_norm_ over the range −0.740 to 1.296 a.u. showing the closest methanol mol­ecules.

**Figure 6:**
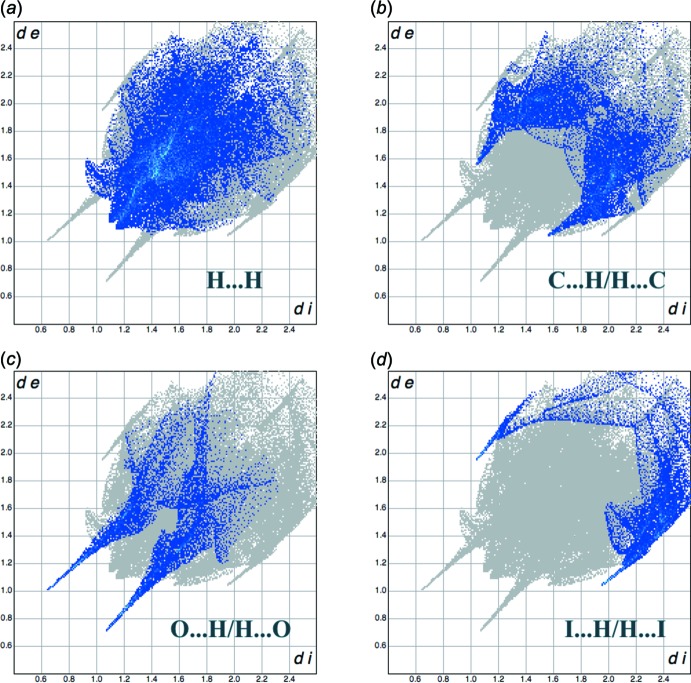
Two-dimensional fingerprint plots delineated into different contact types (*a*)–(*d*) for the title compound. Each blue dot represents a 0.01 Å bin of points on the Hirshfeld surface, with coordinates corresponding to distances (Å) from the points to the nearest inter­ior (*d*_i_) and exterior (*d*_e_) nuclei. Increasing intensity of overlapping points is shown by a colour coding from blue to cyan. The grey background contours correspond to the plot integrated for all contact types.

Additional insight into the crystal packing forces was obtained from a Hirshfeld surface analysis using *CrystalExplorer* (McKinnon *et al.*, 2007 ▸; Spackman & Jayatilaka, 2009 ▸). The largest bright-red spots on the Hirshfeld surface mapped over *d*_norm_ correspond to the (N,O)—H⋯O hydrogen-bonding contacts (Fig. 5 ▸). The pale-red spots are the weaker C⋯H (C18⋯H20), H⋯H (H14*F*⋯H22*B*), I⋯H (I12⋯H21*B*) and I⋯O (I12⋯O23) inter­actions. The most important 2D fingerprint plots, decomposed to highlight particular close contacts of atom pairs and their contribution, are given in Fig. 6 ▸. The relative contributions of the different inter­molecular inter­actions to the Hirshfeld surface area in descending order are: H⋯H (38.2%), C⋯H/H⋯C (20.6%), O⋯H/H⋯O (11.1%), I⋯H/H⋯I (9.7%), N⋯H/H⋯N (7.2%) and C⋯C (5.7%). Contributions from the inter­molecular non- or low-polar inter­actions are much greater than the contributions from the O⋯H contacts. The weak I⋯H inter­actions contribute significantly to the crystal packing.

## Database survey   

**Figure 7:**
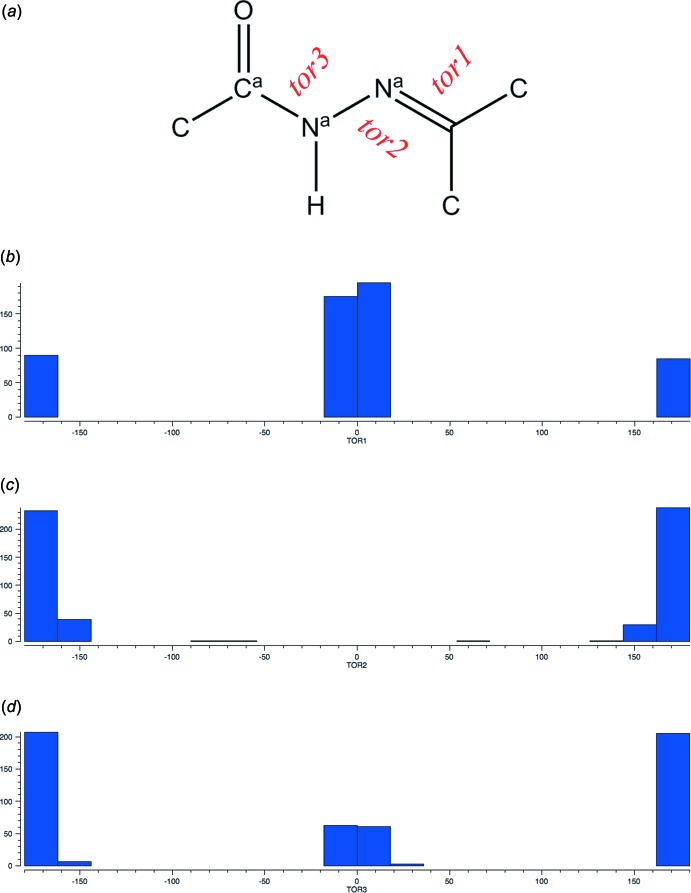
(*a*) The *N*-substituted hydrazide fragment used for a search in the CSD (^*a*^ refers to acyclic). (*b*)–(*d*) Histograms of torsion angles *tor1*, *tor2* and *tor3*, respectively.

A search of the Cambridge Structural Database (CSD, Version 5.39, last update November 2017; Groom *et al.*, 2016 ▸) for the central *N*-substituted hydrazide moiety (Fig. 7 ▸ *a*) resulted in 461 hits. The histograms of the torsion angles show the distribution for torsion angles *tor1* (Fig. 7 ▸ *b*) and *tor3* (Fig. 7 ▸ *d*) as expected for a planar conjugated system. However, the histogram of torsion angle *tor2* (Fig. 7 ▸ *c*) shows the presence of three non-planar entries with torsion angle values of −72.1 (refcode XIJTAN; Buzykin *et al.*, 2012 ▸), −67.9 (refcode NIZTUM; Muniz-Miranda *et al.*, 2008 ▸) and +68.6° (XIJTAN; Buzykin *et al.*, 2012 ▸).

## Synthesis and crystallization   

**Figure 8:**
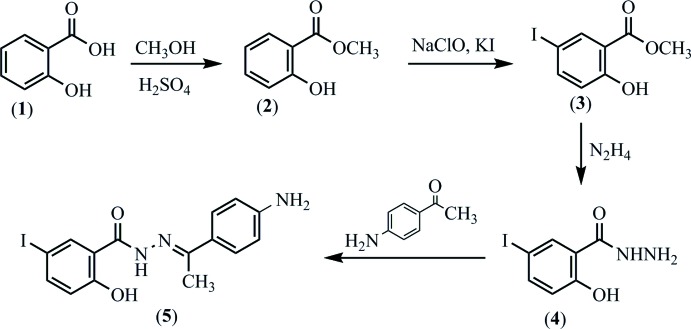
Reaction scheme for the title compound.

The reaction scheme used to synthesize the title compound, **5**, is shown in Fig. 8 ▸. Methyl salicylate, methyl 2-hy­droxy-5-iodo­benzoate and 2-hy­droxy-5-iodo­benzohydrazide were prepared from salicylic acid according to the method described in our earlier work (Nguyen *et al.*, 2012 ▸).

Methyl salicylate, **2**: liquid; b.p. 494-495 K, yield 73%.

Methyl 2-hy­droxy-5-iodo­benzoate (methyl 5-iodo­salicylate), **3**: white needles, m.p. 347–348 K, yield 85%; IR (ν, cm^−1^): 3156, 3080, 2949, 1676, 1604, 527.

2-Hy­droxy-5-iodo­benzohydrazide, **4**: white needles, m.p. 451 K, yield 79%; IR (ν, cm^−1^): 3405, 3322, 1626, 1574, 529; ^1^H NMR (δ, ppm): 12.41 (1H, *br*, OH), 10.12 (1H, *br*, NH), 8.12 (1H, *d*, ^4^ *J* = 2.0, ArH), 7.65 (1H, *dd*, ^3^ *J* = 9.0 Hz, ^4^ *J* = 2.0 Hz, ArH), 6.75 (1H, *d*, ^3^ *J* = 9.0 Hz, ArH), 4.80 (2H, *br*, NH_2_); ^13^C NMR: 166.1 (CO), 158.9, 141.3, 135.5, 119.9, 117.4, 80.5.

(*E*)*-N*’-[1-(4-amino­phen­yl)ethyl­idene]-2-hy­droxy-5-iodo­benzohydrazide, **5**: A solution of 2-hy­droxy-5-iodo­benzohydrazide **4** and 4′-amino­aceto­phenone was refluxed for 2 h. The reaction mixture was cooled down to room temperature and the precipitate obtained was filtered off and crystallized from methanol to give **5** as yellow crystals in 78% yield. M.p. 515–516 K. IR (ν, cm^−1^): 3440, 3298, 3201 (OH, N—H), 2932 (C*sp* ^3^—H), 1634, 1577 (C=O, C=N); ^1^H NMR (δ, ppm and *J*, Hz): 11.11 (1H, *s*, NH), 8.23 (1H, *s*, ArH), 7.70 (1H, *d*, ^3^ *J* = 8.5, ArH), 7.59 (2H, *d*, ^3^ *J* = 8.5, ArH), 6.86 (1H, *d*, ^3^ *J* = 8.5, ArH), 6.59 (2H, *d*, ^3^ *J* = 8.5, ArH), 5.55 (2H, *br*, NH_2_), 2.22 (3H, *s*, –CH_3_); ^13^C NMR (δ, ppm): 161.1 (C=O), 157.0, 154.8, 150.9, 141.6, 138.7, 128.3, 125.2, 121.0, 120.1, 113.7, 82.0, 14.1; MS: *m*/*z* 396.0069 (*M*+H)^+^, calculated for C_15_H_15_IN_3_O_2_: 396.0209.

## Refinement   

Crystal data, data collection and structure refinement details are summarized in Table 2 ▸. The H atoms attached to atoms N2, N21, O11 and O23 were found in a difference-Fourier map and refined freely. The other H atoms were placed at calculated positions and refined in riding mode, with C—H distances of 0.95 (aromatic) and 0.98 Å (CH_3_), and isotropic displacement parameters equal to 1.2*U*_eq_ of the parent atoms (1.5*U*_eq_ for CH_3_). The difference-Fourier map indicated disorder for the H atoms of methyl group C14. The final occupancy factors for the two sets of H atoms are 0.66 (2) and 0.34 (2). In the final cycles of refinement, two reflections showing very poor agreement were omitted as outliers.

## Supplementary Material

Crystal structure: contains datablock(s) I. DOI: 10.1107/S2056989018008204/sj5557sup1.cif

Structure factors: contains datablock(s) I. DOI: 10.1107/S2056989018008204/sj5557Isup2.hkl

Supporting information file. DOI: 10.1107/S2056989018008204/sj5557Isup3.cml

CCDC reference: 1846971

Additional supporting information:  crystallographic information; 3D view; checkCIF report

## Figures and Tables

**Table 1:** Hydrogen-bond geometry (Å, °)

*D*—H⋯*A*	*D*—H	H⋯*A*	*D*⋯*A*	*D*—H⋯*A*
O23—H23⋯O4	0.80 (2)	1.97 (2)	2.7561 (18)	170 (3)
N2—H2⋯O11	0.82 (3)	2.02 (2)	2.665 (2)	134.4 (19)
O11—H11⋯O23^i^	0.76 (3)	1.88 (3)	2.6323 (18)	172 (2)
N21—H21*A*⋯O4^ii^	0.85 (2)	2.14 (2)	2.961 (2)	164 (2)

Symmetry codes: (i) 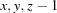; (ii) 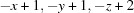.

**Table 2:** Experimental details

Crystal data	
Chemical formula	C_15_H_14_IN_3_O_2_·CH_4_O
*M*_r_	427.23
Crystal system, space group	Monoclinic, *P*2_1_/*c*
Temperature (K)	100
*a*, *b*, *c* (Å)	12.9877 (10), 14.8982 (10), 8.5593 (6)
β (°)	91.806 (2)
*V* (Å^3^)	1655.3 (2)
*Z*	4
Radiation type	Mo *K*α
μ (mm^−1^)	1.95
Crystal size (mm)	0.41 × 0.27 × 0.22

Data collection	
Diffractometer	Bruker D8 Quest CMOS
Absorption correction	Multi-scan (*SADABS*; Bruker, 2014 ▸)
*T*_min_, *T*_max_	0.613, 0.746
No. of measured, independent and observed [*I* > 2σ(*I*)] reflections	45415, 3394, 3086
*R*_int_	0.044
(sin θ/λ)_max_ (Å^−1^)	0.625

Refinement	
*R*[*F* ^2^ > 2σ(*F* ^2^)], *wR*(*F* ^2^), *S*	0.017, 0.041, 1.06
No. of reflections	3394
No. of parameters	231
No. of restraints	1
H-atom treatment	H atoms treated by a mixture of independent and constrained refinement
Δρ_max_, Δρ_min_ (e Å^−3^)	0.61, −0.24

Computer programs: *APEX2* and *SAINT* (Bruker, 2013 ▸), *SHELXS* (Sheldrick, 2008 ▸), *SHELXL2016* (Sheldrick, 2015 ▸) and *OLEX2* (Dolomanov *et al.*, 2009 ▸).

## supplementary crystallographic information

### Crystal data

**Table:**

C_15_H_14_IN_3_O_2_·CH_4_O	*F*(000) = 848
*M**_r_* = 427.23	*D*_x_ = 1.714 Mg m^−^^3^
Monoclinic, *P*2_1_/*c*	Mo *K*α radiation, λ = 0.71073 Å
*a* = 12.9877 (10) Å	Cell parameters from 9842 reflections
*b* = 14.8982 (10) Å	θ = 3.1–30.5°
*c* = 8.5593 (6) Å	µ = 1.95 mm^−^^1^
β = 91.806 (2)°	*T* = 100 K
*V* = 1655.3 (2) Å^3^	Block, yellow
*Z* = 4	0.41 × 0.27 × 0.22 mm

### Data collection

**Table:**

Bruker D8 Quest CMOS diffractometer	3086 reflections with *I* > 2σ(*I*)
φ and ω scans	*R*_int_ = 0.044
Absorption correction: multi-scan (SADABS; Bruker, 2014)	θ_max_ = 26.4°, θ_min_ = 3.1°
*T*_min_ = 0.613, *T*_max_ = 0.746	*h* = −16→16
45415 measured reflections	*k* = −18→18
3394 independent reflections	*l* = −10→10

### Refinement

**Table:**

Refinement on *F*^2^	Primary atom site location: structure-invariant direct methods
Least-squares matrix: full	Hydrogen site location: mixed
*R*[*F*^2^ > 2σ(*F*^2^)] = 0.017	H atoms treated by a mixture of independent and constrained refinement
*wR*(*F*^2^) = 0.041	*w* = 1/[σ^2^(*F*_o_^2^) + (0.0145*P*)^2^ + 1.4435*P*] where *P* = (*F*_o_^2^ + 2*F*_c_^2^)/3
*S* = 1.06	(Δ/σ)_max_ = 0.001
3394 reflections	Δρ_max_ = 0.61 e Å^−^^3^
231 parameters	Δρ_min_ = −0.24 e Å^−^^3^
1 restraint	

### Special details

**Table:**

Geometry. All esds (except the esd in the dihedral angle between two l.s. planes) are estimated using the full covariance matrix. The cell esds are taken into account individually in the estimation of esds in distances, angles and torsion angles; correlations between esds in cell parameters are only used when they are defined by crystal symmetry. An approximate (isotropic) treatment of cell esds is used for estimating esds involving l.s. planes.

### Fractional atomic coordinates and isotropic or equivalent isotropic displacement parameters (Å^2^)

**Table:**

	*x*	*y*	*z*	*U*_iso_*/*U*_eq_	Occ. (<1)
N1	0.40946 (11)	0.57600 (10)	0.63743 (17)	0.0163 (3)	
N2	0.33272 (11)	0.55807 (10)	0.52645 (18)	0.0161 (3)	
H2	0.3379 (16)	0.5699 (15)	0.433 (3)	0.023 (6)*	
C3	0.24587 (13)	0.51777 (11)	0.5728 (2)	0.0148 (3)	
O4	0.22915 (10)	0.50292 (9)	0.71249 (14)	0.0199 (3)	
C5	0.17050 (13)	0.48847 (12)	0.4474 (2)	0.0140 (3)	
C6	0.16570 (13)	0.52242 (11)	0.2943 (2)	0.0145 (3)	
C7	0.08776 (14)	0.49318 (12)	0.1909 (2)	0.0170 (4)	
H7	0.081025	0.519641	0.090110	0.020*	
C8	0.01991 (14)	0.42619 (12)	0.2326 (2)	0.0172 (4)	
H8	−0.031928	0.405794	0.160228	0.021*	
C9	0.02861 (13)	0.38912 (12)	0.3817 (2)	0.0150 (3)	
C10	0.10096 (13)	0.42151 (12)	0.48894 (19)	0.0147 (3)	
H10	0.103575	0.398069	0.592210	0.018*	
O11	0.23620 (10)	0.58419 (9)	0.25082 (15)	0.0182 (3)	
H11	0.233 (2)	0.5920 (18)	0.163 (3)	0.042 (8)*	
I12	−0.06829 (2)	0.28322 (2)	0.44470 (2)	0.01876 (5)	
C13	0.49324 (13)	0.61305 (12)	0.5921 (2)	0.0151 (3)	
C14	0.51380 (15)	0.64301 (14)	0.4276 (2)	0.0217 (4)	
H14A	0.454135	0.628694	0.359179	0.033*	0.66 (2)
H14B	0.574751	0.611872	0.390192	0.033*	0.66 (2)
H14C	0.525871	0.707940	0.426604	0.033*	0.66 (2)
H14D	0.506928	0.591666	0.356473	0.033*	0.34 (2)
H14E	0.583785	0.667320	0.423635	0.033*	0.34 (2)
H14F	0.464045	0.689520	0.395866	0.033*	0.34 (2)
C15	0.57516 (13)	0.62386 (12)	0.7151 (2)	0.0151 (3)	
C16	0.56846 (14)	0.57802 (13)	0.8582 (2)	0.0187 (4)	
H16	0.510394	0.540934	0.875386	0.022*	
C17	0.64415 (14)	0.58565 (12)	0.9740 (2)	0.0183 (4)	
H17	0.637198	0.554354	1.069845	0.022*	
C18	0.73134 (13)	0.63918 (12)	0.9518 (2)	0.0156 (3)	
C19	0.73773 (14)	0.68645 (12)	0.8117 (2)	0.0176 (4)	
H19	0.795006	0.724630	0.795497	0.021*	
C20	0.66115 (14)	0.67820 (12)	0.6957 (2)	0.0175 (4)	
H20	0.667530	0.710410	0.600736	0.021*	
N21	0.80719 (13)	0.64660 (12)	1.06816 (19)	0.0195 (3)	
H21A	0.8080 (17)	0.6075 (16)	1.140 (3)	0.025 (6)*	
H21B	0.8644 (19)	0.6701 (16)	1.040 (3)	0.028 (6)*	
C22	0.31350 (17)	0.69396 (14)	0.9344 (3)	0.0285 (5)	
H22A	0.294430	0.732318	0.845110	0.043*	
H22B	0.380013	0.665329	0.916393	0.043*	
H22C	0.318802	0.730503	1.029528	0.043*	
O23	0.23658 (11)	0.62647 (9)	0.95224 (15)	0.0213 (3)	
H23	0.242 (2)	0.5898 (15)	0.885 (3)	0.040 (8)*	

### Atomic displacement parameters (Å^2^)

**Table:**

	*U*^11^	*U*^22^	*U*^33^	*U*^12^	*U*^13^	*U*^23^
N1	0.0147 (7)	0.0195 (8)	0.0143 (7)	−0.0010 (6)	−0.0037 (6)	−0.0009 (6)
N2	0.0166 (8)	0.0217 (8)	0.0096 (7)	−0.0024 (6)	−0.0033 (6)	0.0005 (6)
C3	0.0170 (9)	0.0125 (8)	0.0147 (8)	0.0020 (7)	−0.0012 (7)	−0.0008 (7)
O4	0.0229 (7)	0.0257 (7)	0.0109 (6)	−0.0068 (5)	−0.0023 (5)	0.0014 (5)
C5	0.0140 (8)	0.0150 (8)	0.0128 (8)	0.0023 (7)	−0.0015 (6)	−0.0025 (7)
C6	0.0163 (8)	0.0131 (8)	0.0142 (8)	0.0010 (7)	0.0009 (7)	−0.0009 (7)
C7	0.0216 (9)	0.0175 (9)	0.0117 (8)	0.0021 (7)	−0.0031 (7)	0.0013 (7)
C8	0.0166 (9)	0.0193 (9)	0.0154 (8)	0.0010 (7)	−0.0048 (7)	−0.0025 (7)
C9	0.0135 (8)	0.0141 (8)	0.0175 (9)	0.0002 (7)	0.0009 (7)	−0.0018 (7)
C10	0.0166 (8)	0.0155 (9)	0.0118 (8)	0.0031 (7)	−0.0001 (7)	0.0004 (7)
O11	0.0225 (7)	0.0215 (7)	0.0104 (6)	−0.0055 (5)	−0.0012 (5)	0.0029 (5)
I12	0.01767 (7)	0.01657 (7)	0.02193 (7)	−0.00289 (4)	−0.00078 (4)	0.00057 (5)
C13	0.0166 (9)	0.0131 (8)	0.0155 (8)	0.0020 (7)	0.0000 (7)	−0.0006 (7)
C14	0.0206 (9)	0.0279 (10)	0.0166 (9)	−0.0017 (8)	−0.0009 (7)	0.0053 (8)
C15	0.0143 (8)	0.0154 (8)	0.0155 (8)	0.0018 (7)	0.0000 (7)	−0.0009 (7)
C16	0.0154 (9)	0.0216 (9)	0.0190 (9)	−0.0051 (7)	0.0002 (7)	0.0021 (7)
C17	0.0180 (9)	0.0210 (9)	0.0160 (8)	−0.0022 (7)	0.0009 (7)	0.0026 (7)
C18	0.0149 (8)	0.0157 (9)	0.0162 (8)	0.0027 (7)	−0.0006 (7)	−0.0050 (7)
C19	0.0150 (9)	0.0169 (9)	0.0210 (9)	−0.0027 (7)	0.0012 (7)	0.0005 (7)
C20	0.0187 (9)	0.0172 (9)	0.0168 (9)	0.0007 (7)	0.0022 (7)	0.0017 (7)
N21	0.0170 (8)	0.0230 (9)	0.0182 (8)	−0.0038 (7)	−0.0019 (6)	0.0006 (7)
C22	0.0291 (11)	0.0251 (10)	0.0318 (11)	−0.0050 (9)	0.0107 (9)	−0.0034 (9)
O23	0.0277 (7)	0.0223 (7)	0.0140 (6)	−0.0043 (6)	0.0014 (5)	−0.0005 (6)

### Geometric parameters (Å, º)

**Table:**

N1—N2	1.381 (2)	C14—H14C	0.9800
N1—C13	1.291 (2)	C14—H14D	0.9800
N2—H2	0.82 (2)	C14—H14E	0.9800
N2—C3	1.349 (2)	C14—H14F	0.9800
C3—O4	1.242 (2)	C15—C16	1.407 (2)
C3—C5	1.495 (2)	C15—C20	1.394 (3)
C5—C6	1.404 (2)	C16—H16	0.9500
C5—C10	1.399 (2)	C16—C17	1.379 (3)
C6—C7	1.394 (2)	C17—H17	0.9500
C6—O11	1.359 (2)	C17—C18	1.403 (3)
C7—H7	0.9500	C18—C19	1.396 (3)
C7—C8	1.386 (3)	C18—N21	1.383 (2)
C8—H8	0.9500	C19—H19	0.9500
C8—C9	1.392 (2)	C19—C20	1.388 (3)
C9—C10	1.380 (2)	C20—H20	0.9500
C9—I12	2.0993 (17)	N21—H21A	0.85 (2)
C10—H10	0.9500	N21—H21B	0.86 (2)
O11—H11	0.76 (3)	C22—H22A	0.9800
C13—C14	1.509 (2)	C22—H22B	0.9800
C13—C15	1.482 (2)	C22—H22C	0.9800
C14—H14A	0.9800	C22—O23	1.429 (2)
C14—H14B	0.9800	O23—H23	0.800 (16)
			
C13—N1—N2	118.21 (15)	C13—C14—H14A	109.5
N1—N2—H2	123.2 (15)	C13—C14—H14B	109.5
C3—N2—N1	118.42 (15)	C13—C14—H14C	109.5
C3—N2—H2	118.4 (15)	C13—C14—H14D	109.5
N2—C3—C5	116.98 (15)	C13—C14—H14E	109.5
O4—C3—N2	122.40 (16)	C13—C14—H14F	109.5
O4—C3—C5	120.59 (16)	C16—C15—C13	120.20 (16)
C6—C5—C3	125.00 (16)	C20—C15—C13	122.59 (16)
C10—C5—C3	116.05 (15)	C20—C15—C16	117.21 (16)
C10—C5—C6	118.94 (16)	C15—C16—H16	119.2
H14Aa—C14—H14B	109.5	C17—C16—C15	121.57 (17)
H14Ba—C14—H14C	109.5	C17—C16—H16	119.2
H14Aa—C14—H14C	109.5	C16—C17—H17	119.7
H14Db—C14—H14E	109.5	C16—C17—C18	120.63 (17)
H14Eb—C14—H14F	109.5	C18—C17—H17	119.7
H14Db—C14—H14F	109.5	C19—C18—C17	118.27 (16)
C7—C6—C5	119.36 (16)	N21—C18—C17	120.49 (17)
O11—C6—C5	119.30 (15)	N21—C18—C19	121.22 (17)
O11—C6—C7	121.33 (16)	C18—C19—H19	119.7
C6—C7—H7	119.4	C20—C19—C18	120.60 (17)
C8—C7—C6	121.12 (16)	C20—C19—H19	119.7
C8—C7—H7	119.4	C15—C20—H20	119.2
C7—C8—H8	120.4	C19—C20—C15	121.68 (17)
C7—C8—C9	119.19 (16)	C19—C20—H20	119.2
C9—C8—H8	120.4	C18—N21—H21A	117.5 (16)
C8—C9—I12	120.11 (13)	C18—N21—H21B	115.7 (15)
C10—C9—C8	120.35 (16)	H21A—N21—H21B	119 (2)
C10—C9—I12	119.54 (13)	H22A—C22—H22B	109.5
C5—C10—H10	119.6	H22A—C22—H22C	109.5
C9—C10—C5	120.78 (16)	H22B—C22—H22C	109.5
C9—C10—H10	119.6	O23—C22—H22A	109.5
C6—O11—H11	111 (2)	O23—C22—H22B	109.5
N1—C13—C14	125.66 (16)	O23—C22—H22C	109.5
N1—C13—C15	115.19 (15)	C22—O23—H23	109.2 (19)
C15—C13—C14	119.13 (15)		
			
N1—N2—C3—O4	5.9 (3)	C8—C9—C10—C5	−3.6 (3)
N1—N2—C3—C5	−172.18 (15)	C10—C5—C6—C7	4.3 (3)
N1—C13—C15—C16	13.9 (2)	C10—C5—C6—O11	−176.74 (15)
N1—C13—C15—C20	−166.49 (17)	O11—C6—C7—C8	175.98 (16)
N2—N1—C13—C14	3.0 (3)	I12—C9—C10—C5	176.29 (13)
N2—N1—C13—C15	−175.48 (15)	C13—N1—N2—C3	178.71 (16)
N2—C3—C5—C6	−20.8 (3)	C13—C15—C16—C17	179.02 (17)
N2—C3—C5—C10	158.64 (16)	C13—C15—C20—C19	−179.11 (17)
C3—C5—C6—C7	−176.25 (16)	C14—C13—C15—C16	−164.75 (17)
C3—C5—C6—O11	2.7 (3)	C14—C13—C15—C20	14.9 (3)
C3—C5—C10—C9	−179.52 (15)	C15—C16—C17—C18	−0.6 (3)
O4—C3—C5—C6	161.03 (17)	C16—C15—C20—C19	0.6 (3)
O4—C3—C5—C10	−19.5 (2)	C16—C17—C18—C19	1.9 (3)
C5—C6—C7—C8	−5.1 (3)	C16—C17—C18—N21	−179.92 (17)
C6—C5—C10—C9	0.0 (3)	C17—C18—C19—C20	−2.0 (3)
C6—C7—C8—C9	1.5 (3)	C18—C19—C20—C15	0.8 (3)
C7—C8—C9—C10	2.9 (3)	C20—C15—C16—C17	−0.6 (3)
C7—C8—C9—I12	−177.01 (13)	N21—C18—C19—C20	179.84 (17)

### Hydrogen-bond geometry (Å, º)

**Table:**

*D*—H···*A*	*D*—H	H···*A*	*D*···*A*	*D*—H···*A*
O23—H23···O4	0.80 (2)	1.97 (2)	2.7561 (18)	170 (3)
N2—H2···O11	0.82 (3)	2.02 (2)	2.665 (2)	134.4 (19)
O11—H11···O23^i^	0.76 (3)	1.88 (3)	2.6323 (18)	172 (2)
N21—H21*A*···O4^ii^	0.85 (2)	2.14 (2)	2.961 (2)	164 (2)

Symmetry codes: (i) *x*, *y*, *z*−1; (ii) −*x*+1, −*y*+1, −*z*+2.

## References

[bb1] Abbas, D., Matter, A.-M., Sanaa, Q. B., Sattar, J. A. A.-S. & Ihsan, A. M. A.-A. (2017). *World J. Pharm. Sci.* **5**, 25–28.

[bb2] Asif, M. & Husain, A. (2013). *J. Appl. Chem.* Article ID, 247203.

[bb3] Bruker (2013). *SAINT*. Bruker AXS Inc., Madison, Wisconsin, USA.

[bb4] Bruker (2014). *APEX2* and *SADABS*. Bruker AXS Inc., Madison, Wisconsin, USA.

[bb5] Buzykin, B. I., Nabiullin, V. N., Mironova, E. V., Kostin, A. A., Tatarinov, D. A., Mironov, V. F. & Litvinov, I. A. (2012). *Russ. J. Gen. Chem.* **82**, 1629–1645.

[bb6] Cui, Z., Ito, J., Dohi, H., Amemiya, Y. & Nishida, Y. (2014). *PLoS One*, **9**, e108338. 10.1371/journal.pone.0108338 PMC4178153 25259805

[bb7] Dolomanov, O. V., Bourhis, L. J., Gildea, R. J., Howard, J. A. K. & Puschmann, H. (2009). *J. Appl. Cryst.* **42**, 339–341.

[bb8] Groom, C. R., Bruno, I. J., Lightfoot, M. P. & Ward, S. C. (2016). *Acta Cryst.* B**72**, 171–179. 10.1107/S2052520616003954 PMC4822653 27048719

[bb9] Khan, M. S., Siddiqui, S. P. & Tarannum, N. (2017). *Hygeia. J. D. Med.* **9**, 61–79.

[bb10] Kumar, N. S., Amandoron, E. A., Cherkasov, A., Finlay, B. B., Gong, H., Jackson, L., Kaur, S., Lian, T., Moreau, A., Labrière, C., Reiner, N. E., See, R. H., Strynadka, N. C., Thorson, L., Wong, E. W., Worrall, L., Zoraghi, R. & Young, R. N. (2012). *Bioorg. Med. Chem.* **20**, 7069–7082. 10.1016/j.bmc.2012.10.002 23141418

[bb11] Majumdar, P., Pati, A., Patra, M., Behera, R. K. & Behera, A. K. (2014). *Chem. Rev.* **114**, 2942–2977. 10.1021/cr300122t 24506477

[bb12] McKinnon, J. J., Jayatilaka, D. & Spackman, M. A. (2007). *Chem. Commun.* pp. 3814–3816. 10.1039/b704980c 18217656

[bb13] Muniz-Miranda, M., Pagliai, M., Cardini, G., Messori, L., Bruni, B., Casini, A., Di Vaira, M. & Schettino, V. (2008). *CrystEngComm*, **10**, 416–422.

[bb14] Murty, M. S. R., Penthala, R., Nath, L. R. & Anto, R. J. (2014). *Lett. Drug. Des. Discov.* **11**, 1133–1142.

[bb15] Nguyen, T. C., Nguyen, Q. T., Nguyen, T. M. N. & Nguyen, T. C. (2012). *Vietnam J. Chem.* **50**, 12–15.

[bb16] Sarshira, E. M., Hamada, N. M., Moghazi, Y. M. & Abdelrahman, M. M. (2016). *J. Heterocycl. Chem.* **53**, 1970–1982.

[bb17] Sheldrick, G. M. (2008). *Acta Cryst.* A**64**, 112–122. 10.1107/S0108767307043930 18156677

[bb18] Sheldrick, G. M. (2015). *Acta Cryst.* C**71**, 3–8.

[bb19] Spackman, M. A. & Jayatilaka, D. (2009). *CrystEngComm*, **11**, 19–32.

[bb20] Wodnicka, A., Huzar, E., Krawczyk, M. & Kwiecień, H. (2017). *Pol. J. Chem. Technol.* **19**, 143–148.

